# Biopolymers-Based Materials Containing Silver Nanoparticles as Active Packaging for Food Applications–A Review

**DOI:** 10.3390/ijms21030698

**Published:** 2020-01-21

**Authors:** Karolina Kraśniewska, Sabina Galus, Małgorzata Gniewosz

**Affiliations:** 1 Department of Food Biotechnology and Microbiology, Institute of Food Sciences, Warsaw University of Life Sciences−SGGW, (WULS-SGGW); Nowoursynowska 159c, 02-776 Warsaw, Poland; malgorzata_gniewosz@sggw.pl; 2 Department of Food Engineering and Process Management, Institute of Food Sciences, Warsaw University of Life Sciences-SGGW (WULS-SGGW); Nowoursynowska 159c, 02-776 Warsaw, Poland; sabina_galus@sggw.pl

**Keywords:** biopolymers, silver nanoparticles, edible film and coating, food packaging materials

## Abstract

Packaging is an integral part of food products, allowing the preservation of their quality. It plays an important role, protecting the packed product from external conditions, maintaining food quality, and improving properties of the packaged food during storage. Nevertheless, commonly used packaging based on synthetic non-biodegradable polymers causes serious environmental pollution. Consequently, numerous recent studies have focused on the development of biodegradable packaging materials based on biopolymers. In addition, biopolymers may be classified as active packaging materials, since they have the ability to carry different active substances. This review presents the latest updates on the use of silver nanoparticles in packaging materials based on biopolymers. Silver nanoparticles have become an interesting component of biodegradable biopolymers, mainly due to their antimicrobial properties that allow the development of active food packaging materials to prolong the shelf life of food products. Furthermore, incorporation of silver nanoparticles into biopolymers may lead to the development of materials with improved physical-mechanical properties.

## 1. Introduction

Packaging materials based on natural biopolymers are becoming more and more popular because of their environmentally friendly, non-toxic, biodegradable, or compostable and biocompatible properties [1].

The biopolymers commonly used for obtaining packaging materials are polysaccharides, proteins, and lipids [2]. Those natural polymers present a good film/coating-forming capability with cohesive structure and are able to create a thin protective layer on the food surface. Basically, films and coating obtained with the use of biopolymers could maintain the quality and extend the stability and shelf-life of food products by (a) controlling exchange of moisture, gases, and lipids between food and the external environment, (b) protecting against microbial contamination, (c) preventing losses of desirable compounds such as flavour volatiles. Furthermore, biopolymers materials can serve as carriers of antimicrobial substances, antioxidants, colour and flavour agents, vitamins or other nutrients, thereby improving the sensory properties and nutritional value of the packaged product [3,4,5]. Recently, new research focuses on the possibility of using edible packaging as carriers of probiotic microorganisms. Enriching edible films and coating with living microorganisms not only confers health benefits to consumers but also maintains food quality and safety by inhibiting the growth of spoilage or pathogenic microorganisms since they produced antimicrobial metabolites [6].

Although biopolymer packaging materials are commonly offered as an alternative to synthetic packaging, so far, they have usually been commercially applied as a complement in the standard packaging system. Limitations of their use are usually associated with weak mechanical properties of biopolymers and their sensitivity to moisture. Therefore, many studies have considered reinforcement of the biopolymer matrix by nanoparticles since they have become a promising option to improve various functional properties of packaging materials [3,7,8,9]. Particularly important nanoparticles, which are considered as new compounds incorporated into a polymer matrix to create innovative nanocomposites materials in food packaging, are silver nanoparticles (AgNPs). Additionally, AgNPs demonstrate good antimicrobial properties against a wide range of microorganisms, including bacteria, yeast, and mould. Therefore, use of such materials in the food industry may be useful for extending the shelf life of food products by preventing the growth of spoilage and pathogenic microflora [3,7,10]. Besides their good antimicrobial activity, the advantages of using silver nanoparticles in edible packaging are also associated with low effects on the sensory attributes in food. Such packaging does not adversely affect the sensory characteristic of food products and makes them more acceptable by consumers. In comparison, natural compounds commonly used in edible packaging such as essential oils or plant extracts due to their strong odor and flavor may strongly modify organoleptic properties of food products, which in turn may limit their use [11].

This overview presents the state of the art concerning possibilities to incorporate silver nanoparticles into biopolymers serving as biodegradable films and coating materials used in food packaging. The paper summarizes the main characteristics and techniques to obtain such food packaging materials, and also describes their antimicrobial, physical, and mechanical properties. In addition, the review discusses the practical application of these packaging nanomaterials in the food industry. Finally, the paper surveys the latest research results associated with the migration of nanoparticles from films and coatings into food and considers the consumer concerns related to the use of such nanomaterials for food packaging application.

## 2. Antimicrobial Properties of Edible Films and Coatings with Incorporated Silver Nanoparticles

Biopolymers have been considered as a suitable medium for the synthesis and stabilization of silver nanoparticles [12,13,14]. This type of polymer-assisted synthesis route provides good dispersion of nanoparticles within a polymer matrix, which subsequently influences the final structural stability and homogeneity of the nanocomposite film, leading to maintenance of strong antimicrobial properties in nanocomposite films [15,16,17].

Techniques in producing nanocomposite films depend on where the nanoparticles are synthesized. Consequently, the preparation of these films is divided into two methods: first, in-situ, where polymeric matrix is used as a reaction medium for formation of silver nanoparticles and acts as a stabilizing agent for them; and second, ex-situ, where polymeric matrix is mainly used as a dispersion and stabilization medium for separately pre-synthesized silver nanoparticles [8].

Bankura et al. [12] found that dextran, a polymer with promising properties used as edible films and coating, can be a medium for in-situ synthesis silver nanoparticles with a size of around 10–60 nm. The authors concluded that silver nanoparticles are well capped with dextran molecules, and thus they can attach to the hydroxyl group of dextran molecules, which prevent their aggregation. Formulations remain stable for more than one month and display effective antibacterial activity against *B. subtilis*, *B. cereus*, *E. coli*, *S. aureus*, and *P. aeruginosa*. Also, several studies have been performed for the in-situ generation of highly monodisperse silver nanoparticles in chitosan film [18,19]. It was shown that silver nanoparticles in chitosan matrices revealed their good dispersion without formation of agglomeration for nearly three months. Strong affinity between polymeric matrix and metal particles corresponds with amine and hydroxyl groups of chitosan [20]. Due to that, effective antimicrobial activity of such nanocomposite materials was observed against both Gram-positive and Gram-negative bacteria.

The biological properties of nanocomposite films were also confirmed when silver nanoparticles were incorporated ex-situ into polymer matrix. For instance, hydroxypropyl methylcellulose (HPMC) polymer with silver nanofiller has been successfully fabricated based on an ex-situ method. In this case, polymer matrices act as a stabilizer to prevent the agglomeration of nanoparticles and thus the nanomaterial exhibits good antimicrobial activity against various bacterial strains [21]. In another study, the antimicrobial potential of trinary bio-composite film based on HPMC/tragacanth/beeswax with pre-synthesized silver nanoparticles was tested. The nanocomposite film inhibited the growth of several pathogens such as *B. cereus*, *S. aureus*, *S. pneumoniae*, *L. monocytogenes*, *E. coli*, *S.* Typhimurium, *P. aeruginosa*, and *K. pneumonia* [22].

Besides interaction of silver nanoparticles with polymer matrices, some other factors such as particle size, shape, and silver concentration should be taken into account in order to fully exploit the antimicrobial activity of polymeric nanocomposites.

For example, De Moura et al. [21] screened the antimicrobial potential of hydroxypropyl methylcellulose (HPMC) films with different sizes of nanoparticles against *E. coli* and *S. aureus*. The authors found that HPMC films containing silver nanoparticles with a diameter of 41 nm had higher bactericidal activity compared to those with a diameter of 100 nm. This result indicates that the particle size has an obvious effect on these antimicrobial properties. Smaller particles exhibit a higher surface area, thus resulting in stronger interaction between them and microbial cells.

Appropriate content of silver nanoparticles in polymers determines their effectiveness in delay, reduction, or even inhibition of the growth of spoilage and pathogenic microorganisms. Several studies have confirmed that depending on the silver nanoparticles’ concentration, nanocomposite materials exhibited bacteriostatic or bactericidal activity. In particular, a higher concentration of silver nanoparticles is required to achieve higher antimicrobial activity [22,23,24,25,26]. For example, Bahrami et al. [22] and Youssef et al. [26] demonstrated that adding a higher percentage of AgNPs to biopolymer cause greater inhibition zones diameter against tested microorganisms. It is important to note that the antimicrobial effect in these applications is related to the release of nanoparticles from polymer nanocomposites and their interaction with microorganisms by direct contact [27]. In the light of several studies, this interaction is based on the physicochemical interaction between the released silver nanoparticles and the surface of the bacterial cell. Although their mode of action is not fully explained, the main action pathways of silver nanoparticles against bacterial cells are: accumulation on the cell surface and destabilization of the structure of the bacterial membrane by formation of gaps, causing leakage of cytoplasmic components, binding to biomolecules such as nucleic acid, protein, and enzymes inside the cells, which contributes to the damage of their functionality and generation of reactive oxygen species (ROS) [10,28,29].

Furthermore, storage temperature of nanocomposite materials plays an important role since it can limit their antimicrobial stability. Khalaf et al. [30] investigated the inhibitory potency of pullulan-based films containing silver nanoparticles against *S. aureus* and *L. monocytogenes* during 7 weeks of storage at different temperatures: 4, 25, 37, and 55 °C. The authors concluded that in order to maintain sustained bactericidal activity, nanomaterials should be stored at temperatures below 25 °C, as temperatures higher than 25 °C significantly reduced their antimicrobial potency.

Table 1 summarized the antimicrobial activity of various biopolymer-based materials containing AgNPs through different preparation methods routes, as well as the size and concentration of AgNPs used.

## 3. Physical Properties of Edible Films with Incorporated Silver Nanoparticles

The main benefits offered by nanomaterials are increased protection and preservation of food, which can be achieved by improving physical and functional properties of film-forming materials. It is well known that characteristics of biopolymer-based packaging materials are dependent on a large number of different factors. The main ones are those including the original source, structural organization of the polymer chain, processing technology, and degree of cross-linking or crystallinity. Therefore, in the application of edible films and coating for food, it is necessary to take into account both the physical characteristics of biopolymer materials and their functional properties.

The main film-forming materials, polysaccharides, and proteins show a hydrophilic nature which is a challenging approach in the development of coating technology for perishable food products. Therefore, incorporation of lipids into a hydrocolloid matrix is one of the methods of improving their low moisture resistance [43]. Hydrocolloid-based materials exhibit very good oxygen permeability values under dry conditions [44]. However, this capacity changes sharply under wet conditions. Oxygen barrier properties become weaker due to the absorption of water vapour, which leads to a loss of network by plasticization and swelling [45]. In general, proteins are very poor moisture barriers, whereas they show good gas transfer properties. Moreover, their structural stability is a major advantage which helps maintain a continuous structure on the food surface. Many treatments, such as physical, chemical, or enzymatic treatments, have been investigated to improve functional properties of edible films and coatings. Nevertheless, the polymer blending process provides an important approach, because composite materials show the complementary advantages of each component as well as minimizing their disadvantages. In addition, better barrier properties can help in maintaining food quality and increasing shelf life without the addition of chemical preservatives [46].

Nanomaterials can be obtained by different methods, including nanolamination, nanoemulsification, or electrostatic techniques, which have a direct impact on the physical properties of the final structure. However, biopolymers with nanoparticles incorporated through encapsulation techniques can be characterized by the nature of the primarily-used material and depend on the concentration and nature of nanoparticles used in the process. In general, nanocomposites with silver nanoparticles are prepared by blending aqueous solutions using a solvent casting method. In this context, the particle size plays an important role in the application of silver nanoparticles and has a large impact on their efficiency and physical properties of final biomaterials. This is also connected with the concentration and distribution of the nanoparticles in the film-forming solutions and dried films.

In general, biopolymer films are transparent without colour or coloured according to the polymer matrix used. Roy et al. [47], who synthesized silver nanoparticles by a green method and incorporated them into carrageenan, obtained nanocomposite films that were less transparent than neat carrageenan films, with a yellowish-brown colour. The presence of yellow colour in the composite films might be due to the yellow colour of silver nanoparticles, which was also observed by others [22,48,49]. The authors observed that the addition of silver nanoparticles not only affected the colour of the films but also improved the UV light barrier property and thermal stability of nanocomposite films. Rhim et al. [25] noted the changes of colour, from pale brown to dark brown, of agar films depending on the concentration of silver nanoparticles. Orsuwan et al. [31] noted that the colour of composite films based on agar and banana powder changed from yellow to dark brown due to the formation of silver nanoparticles in the film-forming solution. This phenomenon was attributed to the characteristic surface plasmon resonance of nanoparticles and was also affected by the concentration of banana powder. Similarly, much darker pectin films were also observed due to the incorporation of silver nanoparticles into the polymer matrix [37]. However, Ortega et al. [50] obtained colourless nanocomposite starch films, indicating that silver nanoparticles caused a slight increase in film opacity while keeping the UV barrier capacity. Therefore, the colour and opacity of the films could change with the presence and concentration of silver particles, thus affecting the final application for food. High values of opacity of the films may protect the product from UV deterioration, although have a negative effect on film transparency, which is directly connected with food appearance.

The main force between the biopolymer and silver nanoparticles of the nanocomposite film matrix is due to the van der Waals interaction between hydroxyl groups of biopolymer and the partial positive charge of the nanofiller. Generally, the mechanical properties of composite films obtained through the blending process depend on the compounds’ interactions and their miscibility, as well as intermolecular interactions between polymer chains. In addition, structural changes can occur in the film matrix caused by the presence of incorporated substances, leading to the less dense structure, which could facilitate greater interaction between components, more than only hydrogen bonds with water molecules. Shankar et al. [37] found that the presence of silver nanoparticles did not significantly change the mechanical properties of pectin films, but a slight increase in mechanical strength was observed. This was attributed to the interaction formed by the hydrogen bond between AgNPs and pectin polymer, which was also noted in the results of FR-IR. However, the mechanical strength of carrageenan films increased at lower amounts of silver nanoparticles added, and decreased linearly with the increasing concentration. A linear increasing tendency was also observed for elongation at break and a linear decreasing tendency for elastic modulus of analysed films [47]. Decreased values of mechanical strength and elastic modulus caused by addition of silver nanoparticles were found by Ortega et al. [50] for nanocomposite starch films and by Yoksan et al. [51] for chitosan–starch blended films. However, silver nanoparticles did not affect elongation at break, which remained constant, leading to resistant and tough materials. Similar observations were reported for agar films [25], corn starch films [52], and for tragacanth/hydroxypropyl methylcellulose/beeswax edible films [22]. On the other hand, Orsuwan et al. [31] observed a decreasing tendency for both tensile strength and elastic modulus for agar/banana powder composite films due to the addition of silver nanoparticles, whereas elongation at break significantly increased. Usually, increasing film flexibility is observed when mechanical strength is decreased, which was also noted for other nanofilms with incorporated silver nanoparticles [48,53]. The film elasticity is also connected with the presence of plasticizer, mainly glycerol, which plays an important role in mechanical property behaviour. In this context, films stored under high relative humidity conditions present higher moisture contents which results in a plasticizing effect acting as a mobility enhancer. In contrast, de Moura et al. [21] noted a significant increase in tensile strength and decrease in elongation at break of hydroxypropyl methylcellulose due to the addition of silver nanoparticles at both analysed sizes, 41 and 100 µm. This was attributed to the partial replacement of the polymer by nanoparticles in the film matrix and had a higher impact when smaller nanoparticles occurred. In this context, similar observations of improved mechanical resistance due to the incorporation of silver nanoparticles were presented for chitosan nanocomposite films [54,55] and konjac glucomannan-chitosan films prepared with the addition of granular cassava starch [56]. In addition, silver nanoparticles incorporated into hydroxymethyl cellulose/bacterial cellulose nanocrystals enhanced both tensile strength and elongation at break [57]. Moreover, Youssef et al. [17] noted that increasing concentration of silver nanoparticles caused linear enhanced values of the Young modulus of chitosan films prepared in acetic or acid solvent, while preserving the elasticity of films. The results show that silver nanoparticles act as a reinforcing inorganic agent of the biopolymeric matrix, which is also attributed to the physical attraction of the components. In general, the mechanical properties of the composite films containing AgNPs are closely related to the distribution and density of the intra and intermolecular interaction which occur between the biopolymer chains in the film matrix. The improvement or weakness of the mechanical strength of films due to the presence and quantity of AgNPs is attributable to the fact that in some cases, biomaterial nature, their homogeneity, or processing conditions are crucial and affect the structure and character of materials.

Surface hydrophobicity or hydrophilicity of edible films can be measured by different techniques, and contact angle analysis is often used. Decreasing water contact angle values due to the addition of silver nanoparticles were observed for chitosan [51], agar/banana powder films [31], and carrageenan films [47]. All those films were classified as hydrophilic nanomaterials (contact angles below 90°). The inclusion of silver nanoparticles might decrease intermolecular interactions between components, thus lowering the surface tension of the films. However, the contact angle for pectin [37] or banana powder films [31] remained constant after the addition of silver nanoparticles. On the other hand, Rhim et al. [25] noted that the contact angle values increased from 46.9 to 84.7° of the agar films and a linear increasing tendency due to the concentration of silver nanoparticles was observed. The increase in film hydrophobicity was attributed to the inclusion of hydrophobic metallic silver in polymer matrix. Similar observations were made by others [53,56]. The addition of silver nanoparticles was found to significantly reduce the moisture absorption capability of hydroxypropyl methylcellulose reinforced with bacterial cellulose nanocrystals [57].

Several studies have shown that the addition of silver nanoparticles caused a significant reduction in the water vapour permeability values of biopolymer nanofilms [21,22,24,25,50,55,56,58,59]. The decrease in the water vapour permeability of investigated films was attributed to the distribution of silver nanoparticles as a discontinuous phase in the polymer matrix, which affected lower diffusion of water molecules through the films. The authors also indicated that the improved water vapour barrier property could be mainly attributed to the increased tortuosity of the polymeric matrix. However, Roy et al. [47] observed that water vapour permeability increased with increasing silver nanoparticle concentration for carrageenan films. Similar results of a weakened water vapour barrier property due to the addition of silver nanoparticles were obtained by Shankar et al. [49] for agar films. Moreover, Kanmani et al. [48] and Orsuwan et al. [24] found no changes in the water vapour permeability values due to the presence of silver nanoparticles when gelatin nanocomposite films and agar/banana powder films, respectively, were investigated. Similar observations were made by Shankar et al. [37] for pectin films.

The research showed that the treatment of films based on cellulose and collagen derivatives, in the commercial form of sausage casings, in a silver solution leads to a change in their structure by ordering and compaction of microfibers forming the cellular structure of the surface microrelief and the internal layer of the polymer matrix. The new materials, which exhibited high bioactivity to different forms of microorganisms, are non-toxic to humans and the environment. Thus, they are extremely promising for biopackaging [60].

The results discussed indicate that physical properties of biopolymer films with incorporated silver nanoparticles depend not only on the concentration and type of nanofiller, but also on the type of polymer matrix and the preparation methods. In addition, the compatibility between components plays an essential role in the formation of composite structure and, consequently, affects the physical properties of films.

## 4. Practical Application of Edible Coating with Silver Nanoparticles

The practical application of edible coating with incorporated silver nanoparticles has been already tested on several foods such as fruit and vegetables, meat, and cheese. Nevertheless, so far, the studies have been conducted on a laboratory scale.

The use of coating materials on fresh and minimally processed fruit and vegetables as a solution to limit the spoilage and increase the shelf life during post-storage has been tested in multiple studies. In the study conducted by Sharanya et al. [61] it was found that storage time for apples and sapota covered with tested coating was extended to 25 days, and for covered tomato, chili, and aubergine, the storage time was extended to 21, 23, and 30 days, respectively. In another study, Moussa et al. [62] analysed the antifungal effects of chitosan coating impregnated with nanosilver applied on strawberries. Fresh strawberries were artificially inoculated by grey mould *Botrytis cinerea*, which is one of the most important pathogens of soft fruits responsible for early latent infections that damage fruits after ripening and cause great losses in agricultural crops. It has been reported that after 7 days of storage at 25 °C, the fungal decay of coated fruits was around 10% compared to 90% of uncoated ones. Similarly, the application of the chitosan coating containing silver nanoparticles on fresh-cut melon had a greater inhibitory effect on count of mesophiles, psychrophiles, Enterobacteria, yeasts, and moulds compared to the uncoated samples stored at 5 °C during 13 days [63]. Jiang et al. [64] conducted the shelf life study of shiitake mushrooms treated by alginate coating with AgNPs storage at 4 ± 1 °C for 16 days. They observed that storage coating efficiently inhibited the growth of mesophilic, psychrophilic, pseudomonas, yeast, and moulds. Moreover, Costa et al. [65] evaluated the influence of alginate coating loaded with silver-montmorillonite nanoparticles on the shelf life of fresh-cut carrots subsequently stored in polypropylene (PP) bags. They reported that using a combination of active coating with PP bags prolonged the shelf-life of carrots for about 70 days compared with 4 days for the uncoated samples. In another study, Hedayati et al. [66] investigated the efficiency of AgNPs dispersed in gum arabic-based coating to extend the shelf-life of green bell peppers. After 21 days of storage at both tested temperatures 7 °C and 20 °C, the coating significantly inhibited the growth of aerobic microorganisms when compared to uncoated samples. Furthermore, coating application onto peppers surface improved their appearance and delayed microbial decay, which allowed the fruits to still be marketable after 21 days of storage. In the next study conducted by Shah et al. [67], the effects of carboxymethylcellulose and guar gum-based coatings containing silver nanoparticles on the microbial quality of kinnow mandarin stored at different temperatures were studied. It was found that during storage, both coatings only limited the growth count of psychrotrophic aerobic yeast and mould count at 10 °C but completely prevented the growth of these microorganisms at 4 °C. Furthermore, the use of such coatings significantly limited fruit rotting, thus prolonging the shelf life of kinnows to about 120 days at 4 °C as against 60 days for kinnows stored at 10 °C. Also, agar coating enriched with AgNPs extended the shelf life of *Citrus aurantifolia* up to 9 days [68].

Several studies have also demonstrated the use of nanocoating as a promising technology in meat preservation. For instance, Zimoch-Korzycka et al. [69] reported the use of hydrosol based on chitosan, hydroxypropylmethyl cellulose, and nanosilver as a protective layer to control the growth of undesirable bacteria on meat surfaces. The authors showed that after 4 weeks of storage at 4 °C, the reduction of bacteria was around 2.5 log CFU/g compared to the uncoated samples. Morsy et al. [39] ascribed the effect of active pullulan edible packaging with silver nanoparticles to inhibiting food-borne pathogens in vacuum-packaged ready-to-eat turkey deli meat stored in refrigerated conditions. The effectiveness of active coating resulted in a reduction of both *Listeria monocytogenes* and *Staphylococcus aureus* from 7 log CFU/g to below the detection limit during 14 days of storage at 4 °C was shown. More recently, antimicrobial edible coating containing silver nanoparticles was tested in vacuum packaged sausages stored at 10 °C. The authors found that antimicrobial activity of the AgNPs was able to inhibit lactic acid bacteria for 30 days, thus significantly increasing the shelf life of the sausages [70].

It has also been shown that nanomaterials are useful for preventing harmful microflora associated with dairy products. Thus far, several studies have been presented using various biopolymers into which silver nanoparticles were incorporated in order to prevent microbial deterioration of Fior di Latte cheese. Incoronato et al. [42] used agar hydrosol with nanosilver to control the cell loads of spoilage (*Pseudomonas* spp. and coliforms bacteria) and functional dairy microorganisms (lactic acid bacteria and coccus-shaped lactic acid bacteria) on cheese stored at 10 °C. The authors reported that the tested silver-based packaging system effectively inhibited the growth of spoilage bacteria without affecting the functional dairy microorganisms. The shelf life of coated dairy products was prolonged to about 6 days compared to 1.5 days in control samples. Similar results were obtained by Gammariello et al. [71] and Mastromatteo et al. [72], who evaluated the microbial safety of Fior di Latte cheese coated with alginate-based films containing silver nanoparticles and stored under modified atmosphere (MAP). As compared to previous work, in those studies, the researchers also noted that using antimicrobial coating in combination with MAP effectively controlled the growth of spoilage microflora with no influence or a slight influence on the growth of lactic acid bacteria. In conclusion, cheese packaged in active coating and stored under MAP conditions showed increased shelf life compared to the cheese stored in the traditional packaging.

All these studies show that silver nanoparticles as active agents incorporated in the polymer matrix exhibited broad antimicrobial activity against predominantly microorganisms which are responsible for limiting the microbial quality of food products. The benefits of using such active packaging as films and coatings are related to slow and gradual release of antimicrobial agents from the polymer matrix onto the food surface where the majority of contamination by spoilage and pathogenic microorganisms occurs for most products. Moreover, this application could also minimize or greatly reduce the amount of preservatives commonly added directly into the bulk of food [73].

## 5. Release of Silver Nanoparticles from Edible Films and Coatings to Food

As reported in the previous section, active packaging based on biopolymers containing silver nanoparticles has great potential in controlling microbial safety of packaged food. The inhibitory effectiveness of packaging materials is determined by the migration of antimicrobial agents from nanocomposite film into headspace inside the package or directly onto the food surface [74,75]. So far, studies on the level of migration have mainly addressed commercially available food packaging nanomaterial. In particular, research has been conducted on release nanoparticles from such synthetic polymers as polyethylene (PE), low-density polyethylene (LDPE), polypropylene (PP), and polyvinyl chloride (PVC) [27,74,76]. However, little information is available about the migration of silver nanoparticles from biopolymers.

Nair et al. [56] measured the migration of silver nanoparticles from chitosan-konjac glucomannan-cassava starch composite film onto bread samples stored for 14 days. It was found that the release level of nanosilver from tested film to the food samples after storage was around 4.3–4.5 µg L^−1^.

In addition, silver migrations throughout starch-based edible coating applied on sausages to the food matrix have also been analysed by Marchiore et al. [70]. It was found that the concentration of silver nanoparticles, which was determined by ICP-MS, directly in the edible coating solution was 23.47 µg AgNPs mL^−1^, and after application on the sausage surface the retained amount of AgNPs was 5.3 ng AgNPs g^−1^ sausages. Moreover, the authors note that commonly used handling procedures such as washing and cooking before eating could additionally remove AgNPs from the product surface to the level of approximately 4 ng AgNPs g^−1^ sausages.

In both studies the overall migration of active substances was below the total legislative migration limit (0.01 mg/kg food) which is set by commission regulation (EC) No. 450/2009 for “non-authorised substances”. Since silver in nano form is recognised as a “non-authorised substance”, established for packaging materials, it seems that it should be subject to this regulation. However, the following part of the commission regulation clearly states that “new technologies that engineer substances in particle size that exhibit chemical and physical properties that significantly differ from those at a larger scale, for example, nanoparticles, should be assessed on a case-by-case basis as regards their risk until more information is known about such new technology. Therefore, they should not be covered by the functional barrier concept” [77,78].

According to that regulation, there is a need to clarify the overall and specific migration of silver nanoparticles to food products. In the assessment of migration, certain key elements such as concentration of migrant nanoparticles and the type of polymer matrices, as well as the mechanism of migration determined by the diffusion rate, depending on, for example, size and morphology of nanoparticles, but also physical-chemical factors such as time and temperature, should be taken into account [27,79,80,81]. All these elements are crucial to determine the risk assessment to human health on the one hand and maintain microbial safety of packaged food on another.

## 6. Future Potential for Use of Nanocomposites in Food Packaging in the Light of Consumer Concerns

In the coming years, silver nanoparticles could play an important role in designing polymeric materials used in the field of active food packaging. However, the major concern about the use of such of packaging materials is associated with insufficient knowledge about the safety and toxicity of silver nanoparticles. So far, researchers have highlighted that silver nanoparticles can induce toxicity to mammalian cells. For instance, AgNPs induce cytotoxicity, genotoxicity, and an inflammatory response in human cells [82]. However, Regiel-Futura et al. [83] and Jena et al. [84] demonstrated that chitosan was a suitable polymeric matrix for AgNP capping, which could exhibit a great bactericidal and fungicidal potential and reduce or totally eliminate cytotoxic effects.

The risk routes in exposure to nanomaterials are dermal, inhalation, and ingestion. It seems obvious that the primary hazard presented by nanocomposite materials used in food packaging is mainly associated with human ingestion because of the possible risk of migration of nanoparticles from the nanocomposites onto the food surface. Thus, a detailed toxicological analysis is needed in order to clarify the risks involved to human health.

Due to the fact that issues concerning the health and safety of nanomaterials are not fully understood, human safety seems to be one of the basic determinants when using them as food packaging materials. To protect consumers from involuntarily exposure to risk, it is necessary to establish maximum levels of the nanoparticles that can be present in the food. Establishing such regulatory requirements seems to be the greatest challenge currently faced by researchers.

## 7. Conclusions

Biopolymers are a new class of packaging materials which can significantly reduce the consumption of synthetic polymer materials, and thus minimize the amount of waste. Biopolymers have great potential to be modified by silver nanoparticles, thus obtaining functional nanocomposite materials. Incorporation of silver nanoparticles into a polymer network of biopolymers leads to changes in the final structure of the film and an impact on the physical and mechanical properties of edible film. Furthermore, incorporation of AgNPs into edible films promotes their antimicrobial properties. Therefore, they may be used as packaging materials for different types of food in order to protect against growth of microorganisms, thereby increasing product shelf-life.

However, it is still necessary to research the mechanism of migration of silver nanoparticles from bio-based packaging material to the product, as well as their effects on the human body and the natural environment, because it still raises consumers’ concerns about the safety of their use. Therefore, further research is needed to determine the optimum levels of silver nanoparticles that can be safely applied in nanomaterials without adversely affecting human health.

## Figures and Tables

**Table 1:** Antimicrobial activity of various biopolymer-based materials containing silver nanoparticles.

Polymer Matrices	Approach	Size of Silver Nanoparticles (nm)	Concentration of AgNO_3_/AgNPs in Film-Forming Solution	Tested Strains	Antimicrobial Effects of Nanocomposite Films	Reference
agar/banana	in-situ	(a) 100 (b) 150–200	AgNO_3_: 1 mM	*L. monocytogenes* ATCC 15313 *E. coli* O157:H7 ATCC 43895	*L. monocytogenes* (a), (b) bacteriostatic activity *E. coli* (a) bacteriostatic activity (b) bactericidal activity	[31]
agar/banana	in-situ	100–300	AgNO_3_: (a) 0.5 mM (b) 1.0 mM (c) 2.0 mM	*L. monocytogenes* ATCC 15313 *E. coli* O157:H7 ATCC 43895	*L. monocytogenes* (a) no inhibitory activity (b), (c) bacteriostatic activity *E. coli* (a) bacteriostatic activity (b) and (c) bacteriocidal activity	[24]
cellulose	in-situ	10–130	–	*E. coli*	Inhibition zone (mm) *E. coli*: 20–25	[32]
chitosan/HEC	in-situ	–	–	*L. monocytogenes, S. aureus, B. cereus*, *E. coli, S.* Typhimurium	Inhibition zone (mm): *L. monocytogenes*: 10–11 *S. aureus*: 10–11 *B. cereus*: 12 *E. coli*: 12–14 *S.* Typhimurium: 13–15	[17]
chitosan/ fucoidan	in-situ	73 ± 9.54	AgNO_3_: 100 µg/mL	*S. aureus* *E. coli*	Inhibition zone (mm): *S. aureus*: 2 *E. coli*: 3	[16]
chitosan adipate/TiO_2_	in-situ	50–100	AgNO_3_: 10 mg/mL	*E. coli* O157:H7 ATCC 700728	*E. coli* Inhibition zone (mm): 12.2 ± 0.7 Reduction level of bacteria count: 6 log CFU/mL	[20]
chitosan	in-situ	75–250	–	*E. coli*	*E. coli* bactericidal effect	[33]
agar	ex-situ: AgNPs pre-synthesis by chemical methods	21.3–23.8	AgNPs (a) 0.2% (b) 0.5% (c) 1.0% (d) 2%	*L. monocytogenes* ATCC 19111 *E. coli* O157:H7 ATCC 11775	*L. monocytogenes* and *E. coli* (a) and (b) no inhibitory effect; (c) bacteriostatic effect (d) bactericidal effect	[25]
agar	ex-situ: AgNPs commercial product purchased from Sigma Aldrich Ag-Cu	<100	Ag-CuNPs (a) 15 mg (b) 30 mg (c) 60 mg (d) 120 mg	*L. monocytogenes* ATCC 19114 *S.* Typhimurium ATCC 14028	Reduction level of bacteria count *L. monocytogenes* (a) 1.5 log CFU/mL (b) 3.0 log CFU/mL (c) 3.5 log CFU/mL (d) 4.5 log CFU/mL *S.* Typhimurium (a) 2.0 log/mL (b) 3.5 log/mL (c) 4.5 log/mL (d) 6.0 log/mL	[34]
chitosan	ex-situ: AgNPs pre-synthesis by biological methods	10–25	AgNPs: (a) 0.5% (b) 1% (c) 2%	*S. aureus, P. aeruginosa,* *Candida albicans, A. niger*	Inhibition zone (mm): *S. aureus* (a); (b) 10; (c) 18 *P. aeruginosa* (a) 11; (b) 12; (c) 15 *C. albicans* (a) 12; (b) 12; (c) 19 *A. niger* (a) 10; (b) 8; (c) 9	[26]
guar gum	ex-situ: AgNPs commercial product purchased from Sigma Aldrich Ag-Cu	<100	AgNPs: (a) 7.5 mg (b) 15 mg (c) 30 mg	*L. monocytogenes* ATCC 19114 *S.* Typhimurium ATCC 14028	Reduction level of bacteria count *L. monocytogenes* (a) 0.5 log CFU/mL (b) 2.0 log CFU/mL (c) 3.5 log CFU/mL *S.* Typhimurium (a) 1.5 log CFU/mL (b) 2.5 log CFU/mL (c) 4.5 log CFU/mL	[35]
gelatin	ex-situ: AgNPs commercial product purchased from Sigma Aldrich Ag-Cu	<100	AgNPs: (a) 0.5% (b) 1.0% (c) 2.0% (d) 4.0%	*L. monocytogenes* ATCC 19114 *S.* Typhimurium ATCC 14028	Reduction level of bacteria count *L. monocytogenes:* (a)‒(d) 0.5‒3.5 log CFU/mL *S.* Typhimurium (a) no inhibition effect (b)‒(d) reduction upper 7 log CFU/mL	[36]
HPMC	ex-situ: AgNPs pre-synthesis by chemical methods	(a) 41 (b) 100	-	*S. aureus* ATCC 25922 *E. coli* ATCC 25923	Inhibition zone (mm): *S. aureus*: (a) 3.11; (b) 1.35 *E. coli*: (a) 2.75; (b) 1.05	[21]
tragacanth/ HPMC/ beeswax	ex-situ: AgNPs commercial product purchased from US Research Nanomaterials Inc.	8–10	AgNPs: 2, 4, 8%	*L. monocytogenes* ATCC 7644, *S. aureus* ATCC 25923, *B. cereus* ATCC 1247, *S. pneumoniae* ATCC 49615, *E. coli* ATCC 8739, *S.* Typhimurium ATCC 14028, *P. aeruginosa* ATCC 9027, *K. pneumoniae* ATCC10031	observed inhibitory effects against all strains of bacteria; higher percentage of AgNPs into biopolymer matrix caused greater inhibitory zones diameter	[22]
pectin	ex-situ: AgNPs pre-synthesis by biological methods	20–80	AgNPs: 100 µg/mL	*L. monocytogenes* ATCC 15313 *E. coli* O157:H7 ATCC 43895	Zone of inhibition (mm): *L. monocytogenes*: 3.9 *E. coli*: 8.4	[37]
pullulan	ex-situ: AgNPs pre-synthesis by chemical methods	6–18	AgNPs: (a) 0.156 mg/g (b) 0.317 mg/g (c) 0.803 mg/g (d) 1.710 mg/g	*Aspergillus niger*	Fungal growth inhibition (%) (a) 12; (b) 22; (c) 45; (d) 76	[38]
pullulan	ex-situ: AgNPs commercial product purchased from Sigma Aldrich	100	AgNPs: 0.02 (*v/v*)	*L. monocytogenes* ATCC 94229 *S. aureus* ATCC 11988 *E. coli* O157:H7 ATCC 43895 *S.* Typhimurium ATCC 14028	inhibitory effect against *L. monocytogenes* and *S. aureus* was observed no inhibition effect against *E. coli* O157:H7 and *S.* Typhimurium was observed	[39]
pullulan	ex-situ: AgNPs commercial product purchased from Sigma Aldrich	100	AgNPs: 2%	*L. monocytogenes* ATCC 94229 *S. aureus* ATCC 11988	Inhibition zone (mm): *L. monocytogenes*: 25‒30 *S. aureus* 15–23	[30]
pullulan/PVA	ex-situ: AgNPs commercial product purchased from Sigma Aldrich Miji Tech., Korea	15–30	AgNPs: 1–4%	*S. aureus* ATCC 6538 *E. coli* ATCC 25922	bactericidal effect regardless of concentration against both tested strains of bacteria	[15]
sodium alginate	ex-situ: AgNPs pre-synthesis by biological methods	5–40	–	*S. aureus* ATCC 8739 *E. coli* ATCC 6538	inhibitory effect against both tested strains of bacteria was observed	[40]
sodium alginate/ chitosan	ex-situ: AgNPs pre-synthesis by biological methods	5–21	–	*B. cereus* MTCC 1305, *E. faecalis* MTCC 439, *E. coli, E. aerogenes* MTCC 2822, *P. aeruginosa* MTCC 2488	Inhibition zone (mm): *B. cereus:* 6.0 *E. faecalis:* 5.1 *E. coli*: 1.9 *E. aerogenes:* 1.5 *P.aeroginosa:* 3.1	[41]
agar	ex-situ		Ag-MTT NPs (a) 10 mg; (b) 15 mg; (c) 20 mg	*Pseudomonas* spp.	Reduction level of bacteria count (a) 1.88 log CFU/g (b) 2.09 log CFU/g (c) 3.59 log CFU/g	[42]
zein	ex-situ		Ag-MTT NPs (a) 10 mg; (b) 15 mg; (c) 20 mg	*Pseudomonas* spp.	(a) 1.53 log CFU/g (b) 1.83 log CFU/g (c) 2.12 log CFU/g	[23]

AgNO_3_—silver nitrate; AgNPs—silver nanoparticles; HEC—hydroxyl ethyl cellulose; HPMC—hydroksypropyl methylocellulose; PVA—poly (vinyl alcohol).
